# Supplementary material for: Structural basis for the activation of the lipid scramblase TMEM16F
Source: Nat Commun. 2022 Nov 5;13:6692. doi: 10.1038/s41467-022-34497-x (PMC9637102; doi:10.1038/s41467-022-34497-x)
Supplement: Supplementary file 1 — Supplementary Information [file 41467_2022_34497_MOESM1_ESM.pdf]

Supplementary Information

Structural basis for the activation of the lipid scramblase TMEM16F

Melanie Arndt, Carolina Alvadia, Monique S. Straub, Vanessa Clerico Mosina, Cristina Paulino and Raimund Dutzler

Supplementary Figures

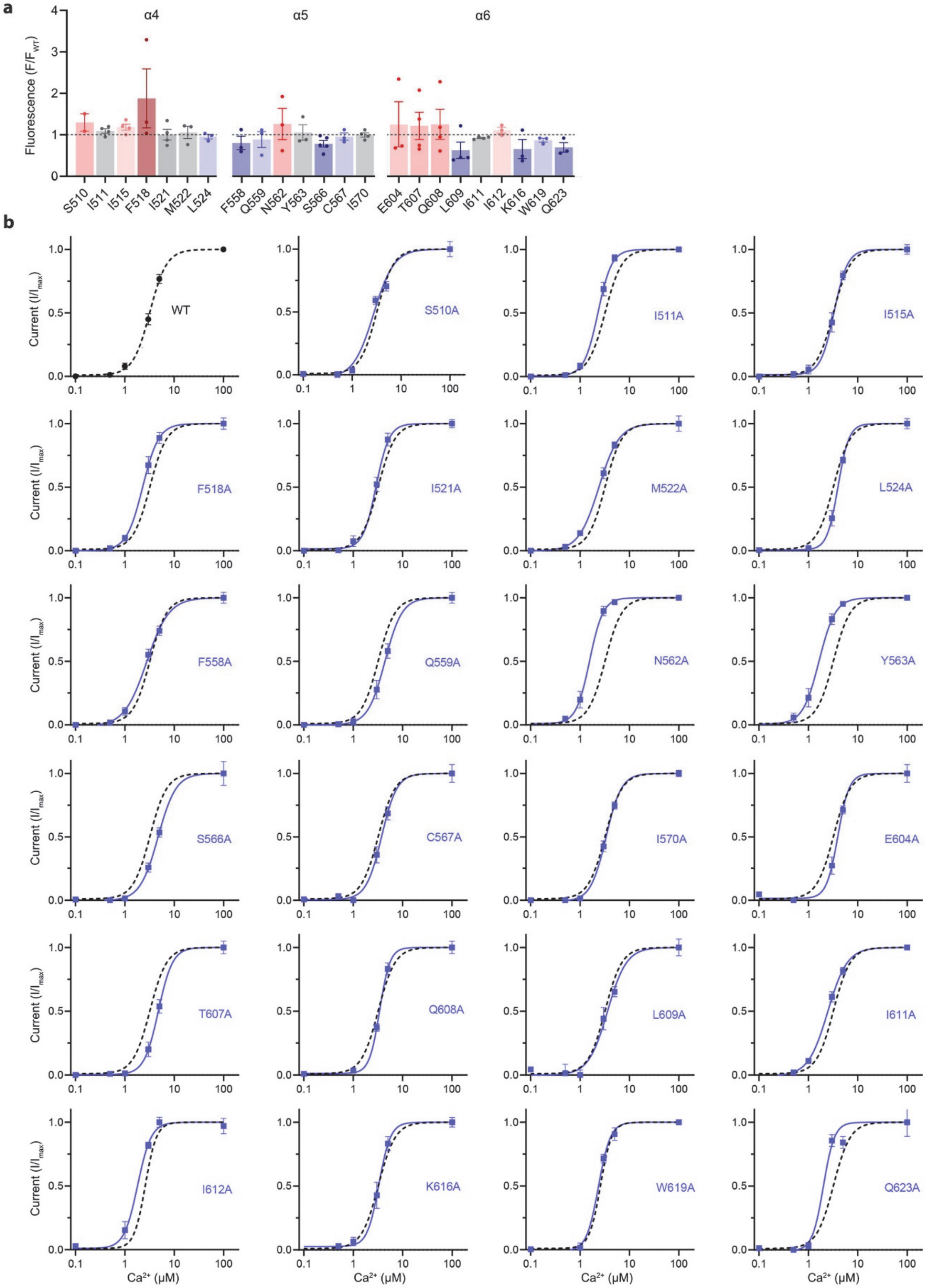

Supplementary Fig. 1: Mutant Screen. a, Fluorescence in a cell-based lipid scrambling assay monitoring the binding of tagged annexin V to phosphatidylserine at the cell surface. Data shows lipid transport activity of TMEM16F alanine point mutants at elevated Ca^{2+}-concentrations (600 seconds

after addition of ionomycin). Values are normalized to WT (dashed line). Data show mean of indicated number of biological replicates (S510A, n=2; F518A, M522A, L524A, F558A, Q559A, N562A, Y563, C567A, E604A, I612A, K616A, W619A, Q623A, n=3; I511A, I515A, I521A, I570A, T607, Q608, L609, I611A, n=4; S566A, n=5) and were obtained from the same recordings as shown in Fig. 1a (top), errors are s.e.m. **b**, Ca^{2+} concentration-response relationships of TMEM16F mutants. Plots show values derived from rundown-corrected currents recorded from transfected HEK293T cells in excised inside-out patches. Data was normalized, lines show fit to a Hill-equation, dashed line refers to WT. Error bars are s.e.m. Datapoints show mean of indicated number of biological replicates (WT n =10, S510A n=7, I511A n=7, I515A n=5, F518A n=5, I521A n=7, L524A n=5, M522A n=5, K530A n=7, F558A n=7, Q559A n=5, N562A n=5, Y563A n=4, S566A n=5, C567A n=5, I570A n=8, E604A n=6, T607A n=7, Q608A n=6, L609A n=4, I611A n=7, I612A n=3, K616A n=4, W619A n=4, Q623A n=7). Source data are provided as a Source Data file.

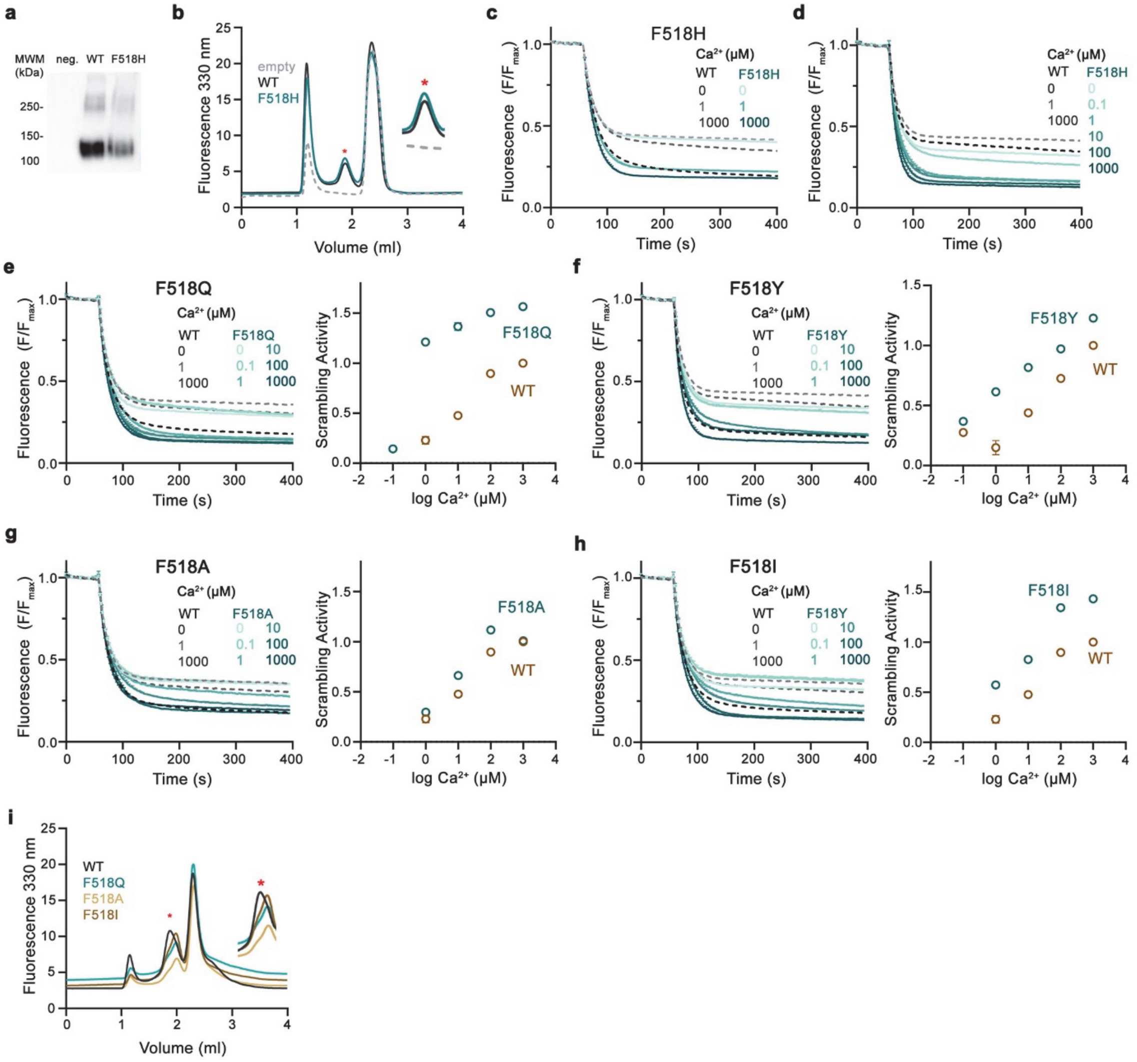

Supplementary Fig. 2: Scrambling activity of TMEM16F mutants. **a**, Western blot showing the protein content of proteoliposomes containing either WT or the mutant F518H. **b**, SEC profile of solubilized proteoliposomes. **c-h** Ca^{2+}-dependent lipid transport of activating TMEM16F mutants recorded with a liposome-based in vitro scrambling assay. Traces show the fluorescence decrease of tail-labeled NBD lipids upon addition of the reducing agent dithionite (at 60 s). Fluorescence levels decreasing below 0.5 indicate lipid scrambling by reconstituted TMEM16F. Data depict the mean of three technical replicates, error bars (s.e.m.) are smaller than the displayed line width. Dashed lines refer to WT at the indicated concentrations. **c**, **d** Data from two different reconstitutions of the TMEM16F mutant F518H. **e-h** Data from reconstitutions of the mutants F518Y (**e**), F518Q (**f**), F518A (**g**) and F518I (**h**). Quantification of the Ca^{2+} concentration-response relationships plotted as $1\text{-}F/F_{wt}^{0Ca2+}$ are shown right. a-c Data was obtained from the same batch of reconstitution. **i**, SEC profiles of solubilized proteoliposomes of indicated TMEM16F constructs. b, i Asterisk indicates peak corresponding to TMEM16F and its mutants. Inset shows blowup of the TMEM16F peak. Source data are provided as a Source Data file.

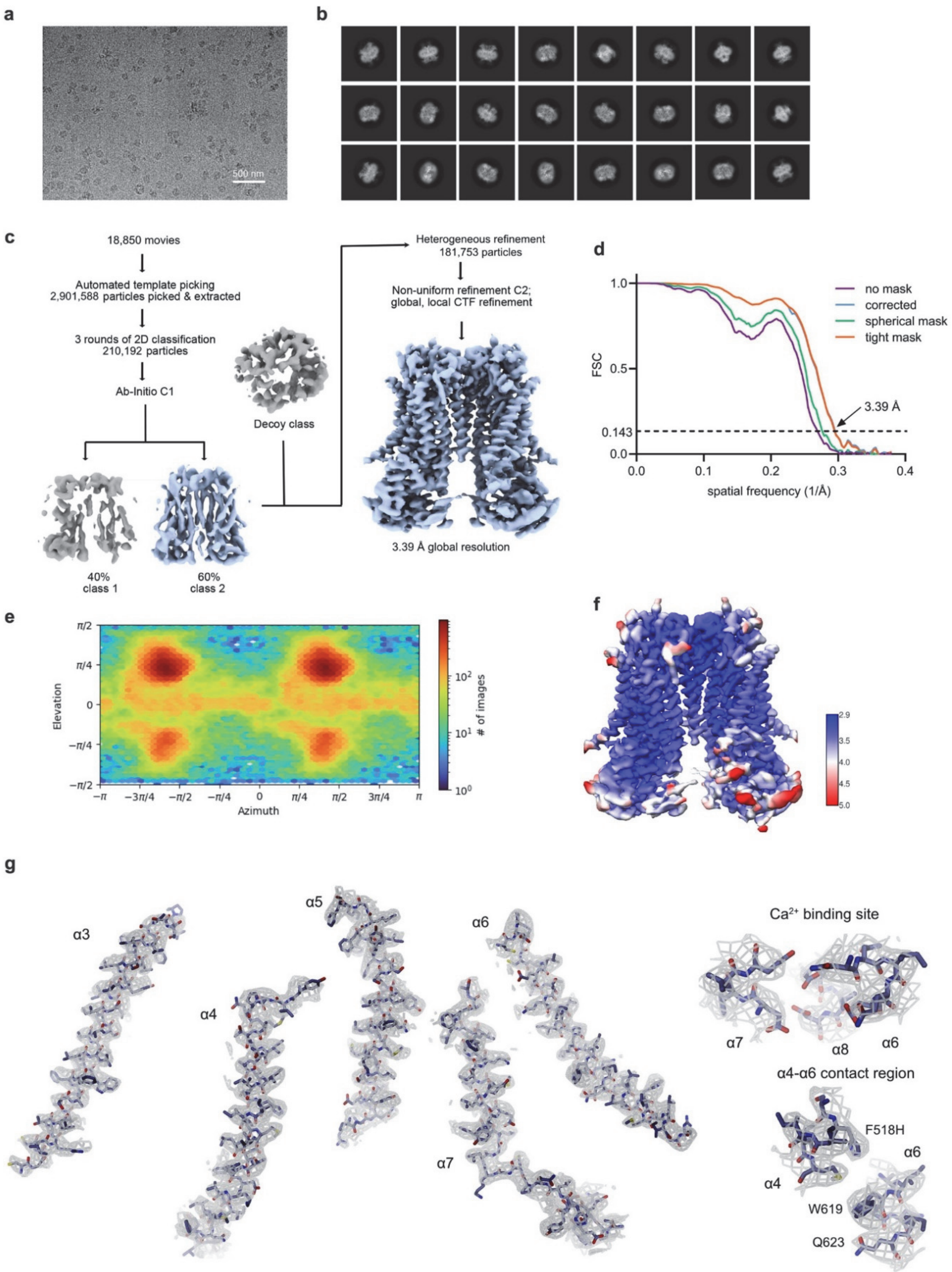

Supplementary Fig. 3: Cryo-EM structure of F518H^{noCa}. a, Representative cryo-EM micrograph. **b**, 2D class averages of TMEM16F F518H^{noCa} in digitonin. **c**, Image and particle processing workflow. **d**, FSC plot used for resolution estimation. **e**, Angular view distribution of the particles contributing to the final 3D volume with C2 symmetry imposed. **f**, Local resolution estimation of the final map. **g**, Cryo-EM densities (contoured at 9.5 σ) superimposed on selected parts of the model.

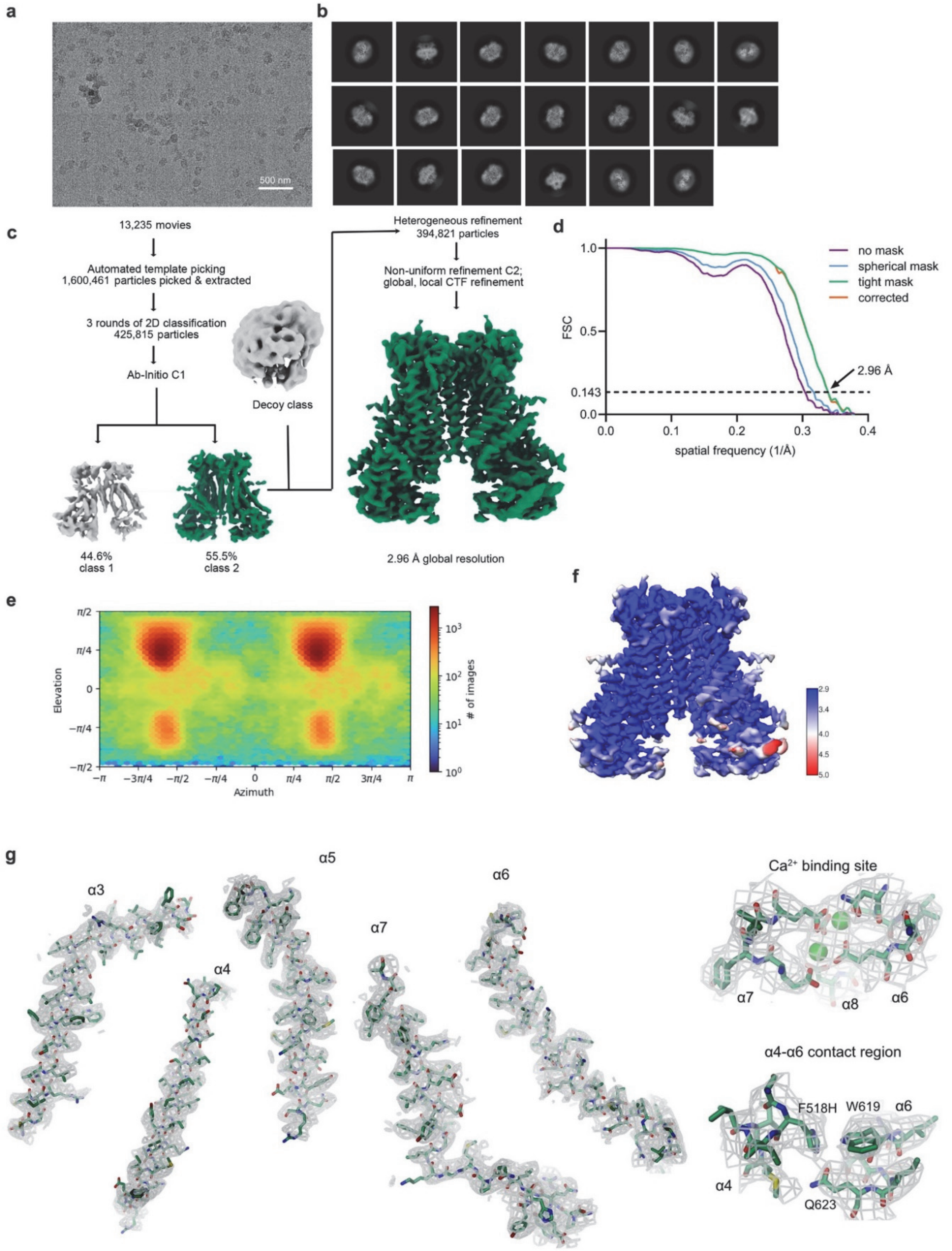

Supplementary Fig. 4: Cryo-EM structure of F518H^{Ca}. a, Representative cryo-EM micrograph and **b**, 2D class averages of TMEM16F F518H^{Ca} in digitonin. **c**, Image and particle processing workflow. **d**, FSC plot used for resolution estimation. **e**, Angular view distribution of the particles contributing to the final 3D volume with C2 symmetry imposed. **f**, Local resolution estimation of the final map. **g**, Cryo-EM densities (contoured at 6.5 σ) superimposed on selected parts of the model.

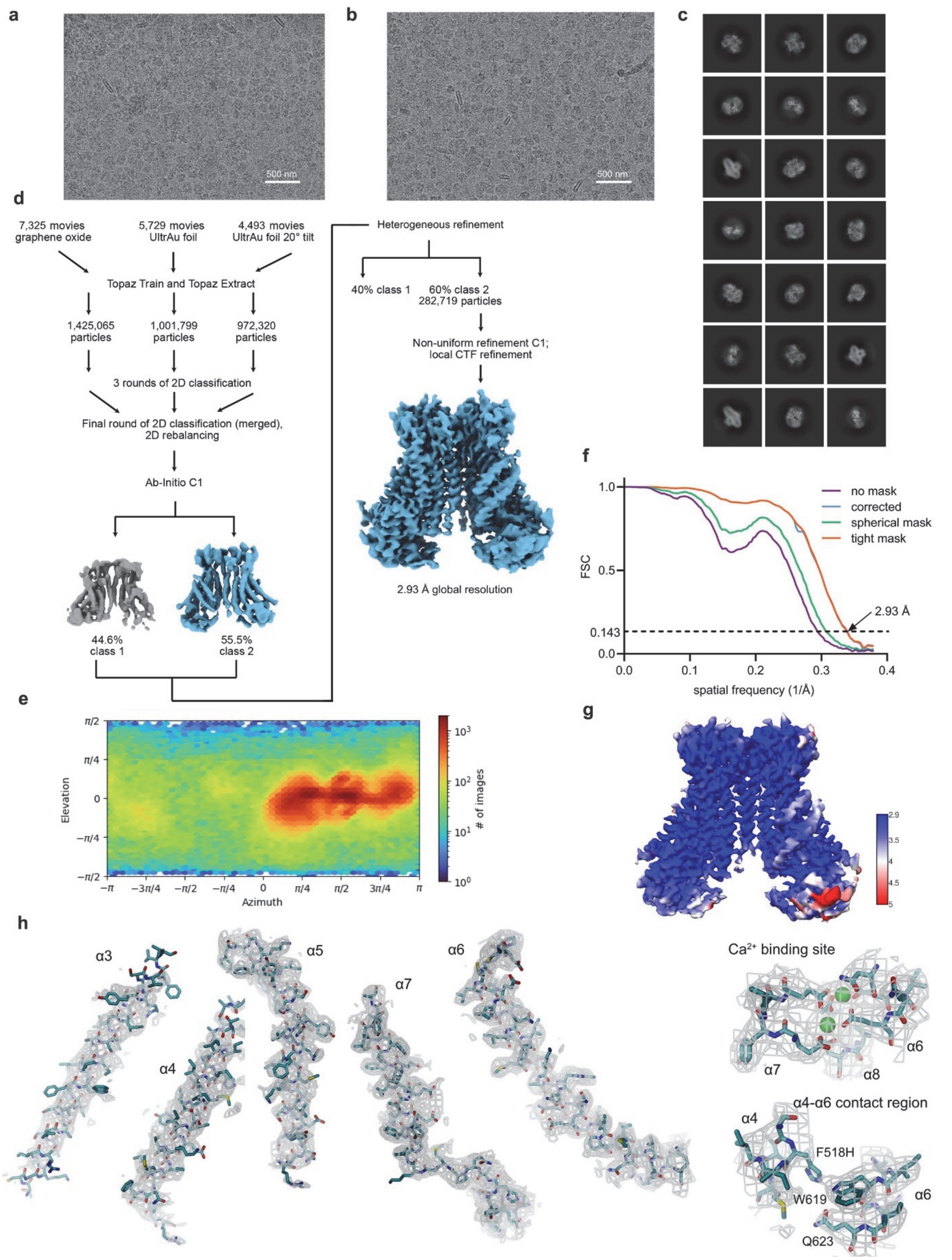

Supplementary Fig. 5: Cryo-EM structure of F518H^{Ca}$_{ND}$. a, Representative cryo-EM micrograph of TMEM16F F518H^{Ca}$_{ND}$ frozen on UltrAuFoil grids (Quantifoil) and **b**, graphene oxide copper grids (EMS Scientific). **c**, Representative 2D-class averages from the combined dataset. **d**, Image and particle processing workflow. **e**, Angular view distribution of the particles contributing to the final 3D volume. **f**,

FSC plot used for resolution estimation. **g**, Local resolution estimation of the final map. **h**, Cryo-EM densities (contoured at 6.5 σ) superimposed on selected parts of the model.

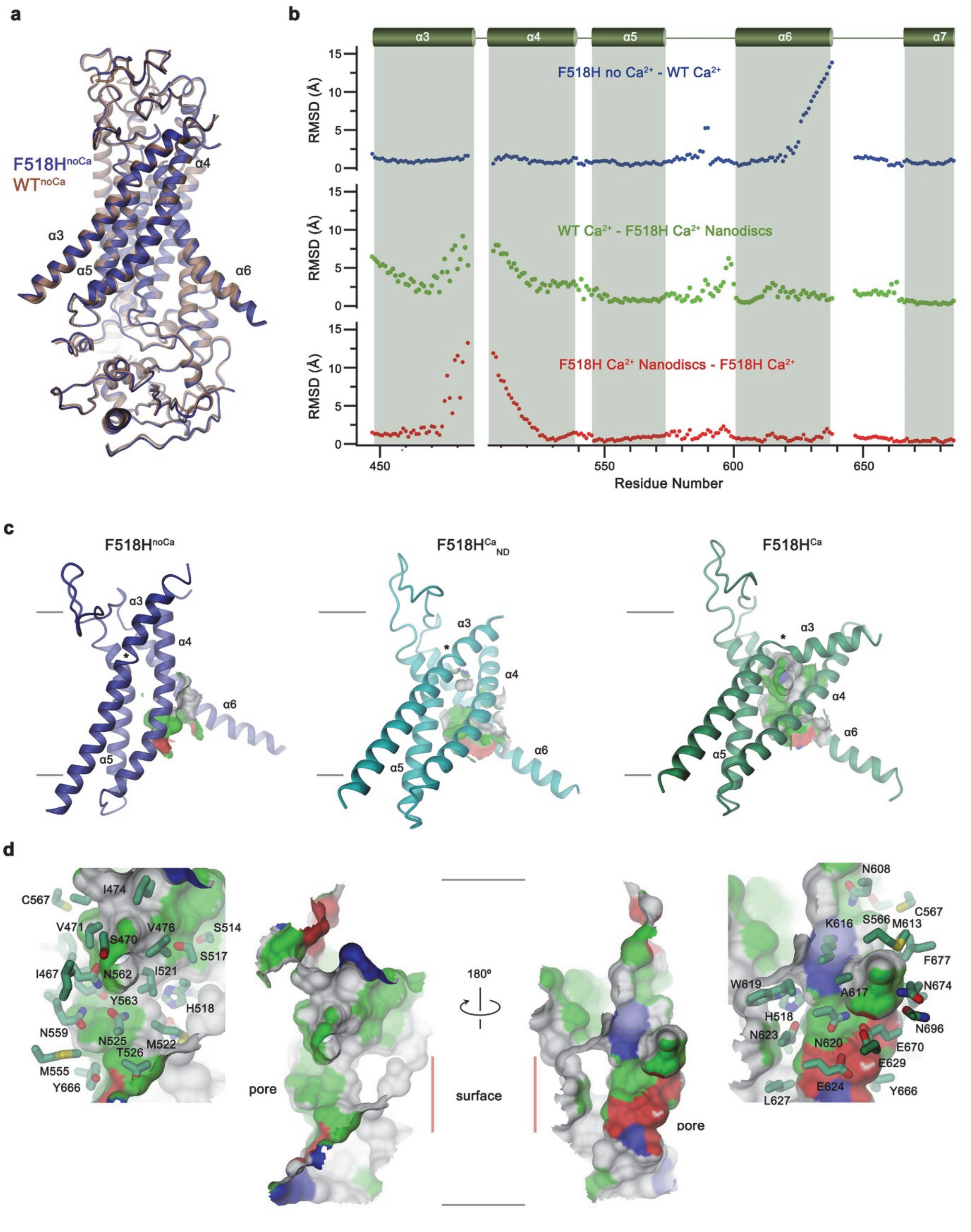

Supplementary Fig. 6: Conformational transitions. a, Superposition of a single subunit of WT^{noCa} (PDBID 6QPB) on F518H^{noCa}. The view is from within the membrane towards the subunit cavity. **b**, Root mean square deviation of Cα positions measured for individual residues of TMEM16F subunits after the

superposition of indicated protein conformations (top F518H^{noCa} superimposed on WT^{Ca}, PDBID 6QP6; center WT^{Ca} superimposed on F518H$^{Ca}_{ND}$; bottom F518H$^{Ca}_{ND}$ superimposed on F518H^{Ca}). Secondary structure elements are shown on top and highlighted as shaded regions. **c**, Progressive opening of an aqueous pore in TMEM16F mutants. The figure displays ribbon models of the pore region in indicated structures. The protein surface around the narrow pore constriction is shown with residues lining the surface colored based on their physico-chemical properties (blue basic, red acidic, green polar). Asterisk indicates location of Gly 473 serving as hinge on α3. Membrane boundaries are indicated. **d**, Pore region as observed in the structure F518H^{Ca}. Shown is the molecular surface around the pore region in two different orientations with the left panels sharing the same orientation as depicted in c. Membrane boundaries are displayed by grey lines. The protein-enclosed pore is labeled (pore). Red line indicates contact region between α4 and α6 shielding the pore from the membrane (surface). Regions above and below are exposed to the bilayer. Coloring of the surface is as in c. Insets (left and right) show close-ups of the pore with sidechains of contacting residues displayed as sticks and labeled.

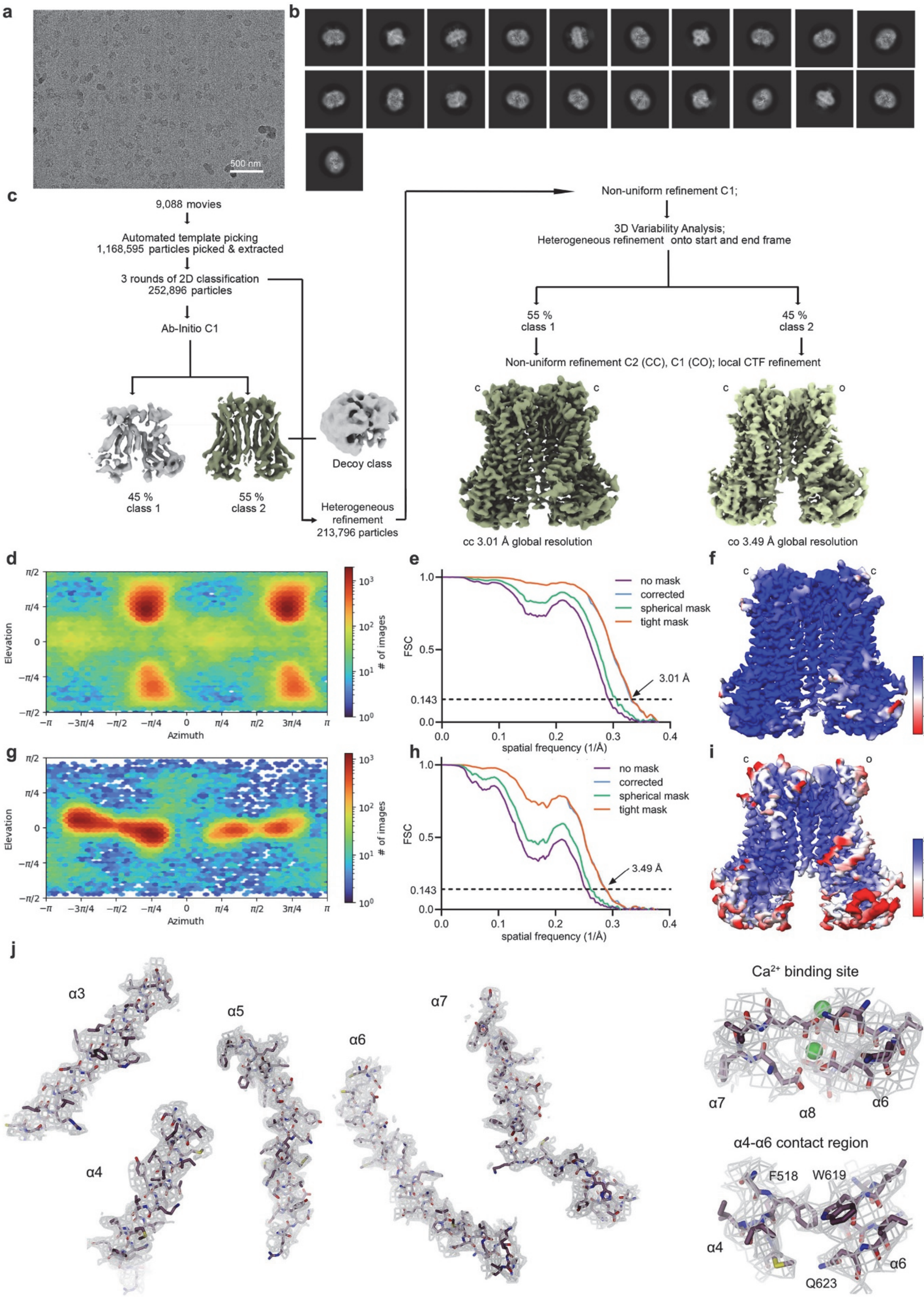

Supplementary Fig. 7: Cryo-EM structure of N562A^{Ca}. a, Representative cryo-EM micrograph and **b**, 2D-class averages of TMEM16F N562A^{Ca}. **c**, Image and particle processing workflow. **d**, Angular view distribution of the particles contributing to the final 3D volume of the symmetric population

$N562A^{Ca}_{cc}$ where both subunits adopt a closed (c) conformation, with C2 symmetry imposed. **e**, FSC plots used for resolution estimation of $N562A^{Ca}_{cc}$. **f**, Local resolution estimation of the final map of $N562A^{Ca}_{cc}$. **g**, Angular view distribution of the particles contributing to the final 3D volume of the asymmetric population $N562A^{Ca}_{oc}$, with subunits adopting closed (c) and open (o) conformations, processed without application of rotational symmetry (C1). **h**, plots used for resolution estimation of $N562A^{Ca}_{co}$ (h). **i**, Local resolution estimation of the final map of $N562A^{Ca}_{co}$. **j**, Cryo-EM densities of the protein in an open conformation (contoured at 6.5 σ) superimposed on selected parts of the model.

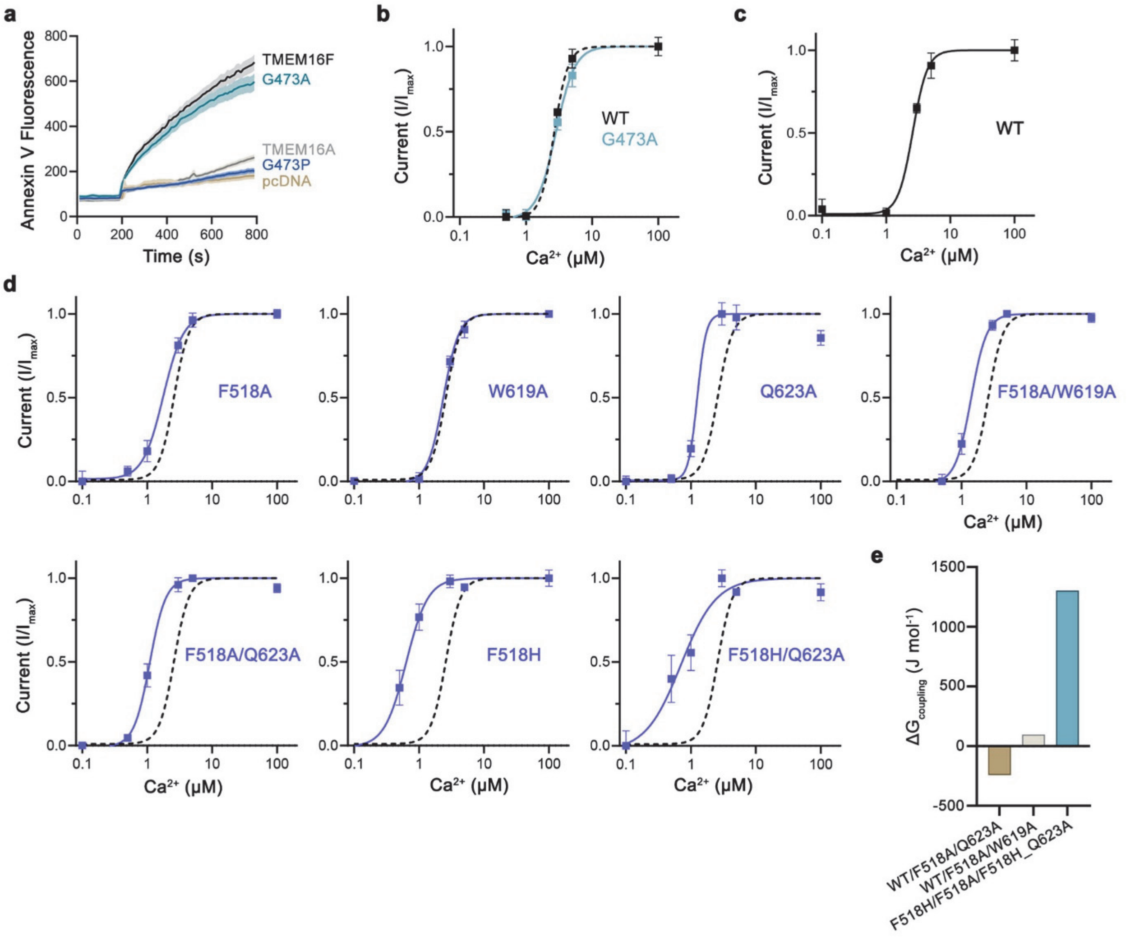

Supplementary Fig. 8: Functional characterization of structure-based mutants. a, Representative annexin V fluorescence traces for different constructs obtained from the cellular scrambling assay. The intracellular Ca^{2+} concentration was increased by addition of ionomycin 180 s after the initiation of the recording. Data show mean of transfected cells within one well from a single replicate (pcDNA n=81, TMEM16A n=193, TMEM16F n=141, TMEM16F G473A n=147, TMEM16F G473P n=215), errors are s.e.m. **b-d**, Concentration-response relationships for Ca^{2+}-activated currents of TMEM16F mutants of **b**, the α3 hinge residue mutant G473A (WT n=8, G473A n=4), **c**, WT (n=5) and **d**, mutants of residues in the subunit cavity used for double mutant cycle analysis (displayed in Fig. 8f, F518A n=10, W619A n=4, Q623A n=4, F518H n=2, F518A Q623A n=10, F518A W619A n=11, F518H Q623A n=5). Plots show data from rundown corrected currents recorded from transfected HEK293T cells in excised inside-out patches. Data was normalized, lines show fit to a Hill-equation not constraining the Hill coefficient. Dashed line refers to WT as displayed in **c**. **b-d**. Datapoints show mean of multiple experiments recorded from n independent cells, error bars are s.e.m. **e**, Coupling energies between selected residues obtained from data displayed in **d**. Energies were calculated as in Fig. 8f except that the Hill coefficient for the fit was fixed at a value of 2. WT/F518A/Q623A and WT/F518A/W619A refer to the cycle quantifying the effect of mutations F518A and either Q623A or W619A relative to WT, F518H/F518A/F518H_Q623A to the cycle of F518A and F518H_Q623A relative to the mutant F518H. Source data are provided as a Source data file.

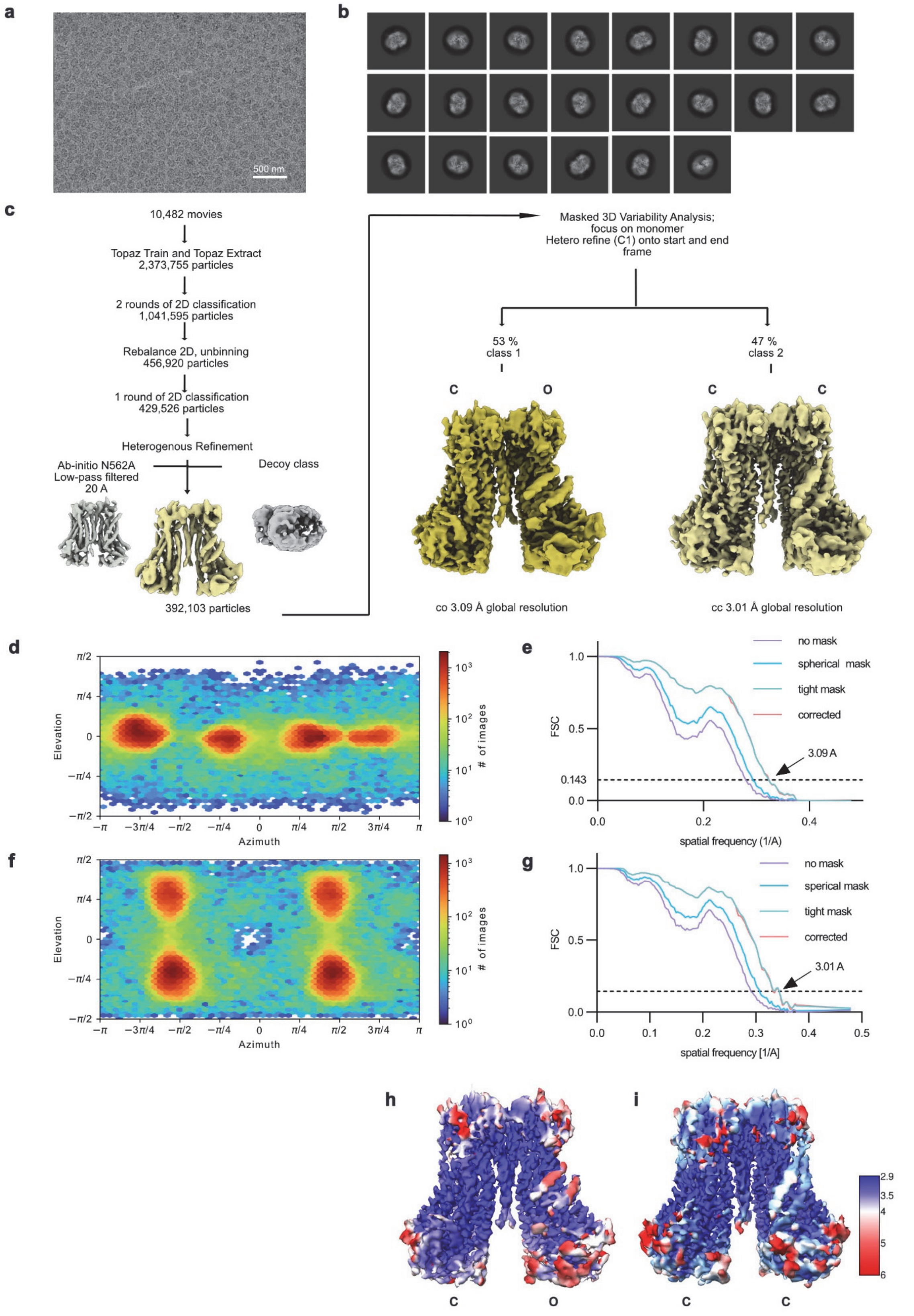

Supplementary Fig. 9: Cryo-EM structure of F518A_Q623A^{Ca}. a, Representative cryo-EM micrograph and **b**, 2D-class averages of TMEM16F F518A_Q623A^{Ca}. **c**, Image and particle processing

workflow. **d**, Angular view distribution of the particles contributing to the final 3D volume of the asymmetric population F518A_Q623A$^{Ca}_{co}$ where one subunit adapts a closed (c) and one subunit adapts an open state (o), processed without application of rotational symmetry. **e**, FSC plots used for resolution estimation of F518A_Q623A$^{Ca}_{cc}$. **f**, Angular view distribution of the particles contributing to the final 3D volume of the symmetric population F518A_Q623A$^{Ca}_{cc}$, with both subunits adopting a closed (c) conformation, processed with application of C2 symmetry. **f**, FSC plots used for resolution estimation of F518A_Q623A$^{Ca}_{cc}$. **h**, **i**, Local resolution estimations of the final maps of F518A_Q623A$^{Ca}_{co}$ (**h**) and F518A_Q623A$^{Ca}_{cc}$ (**i**).

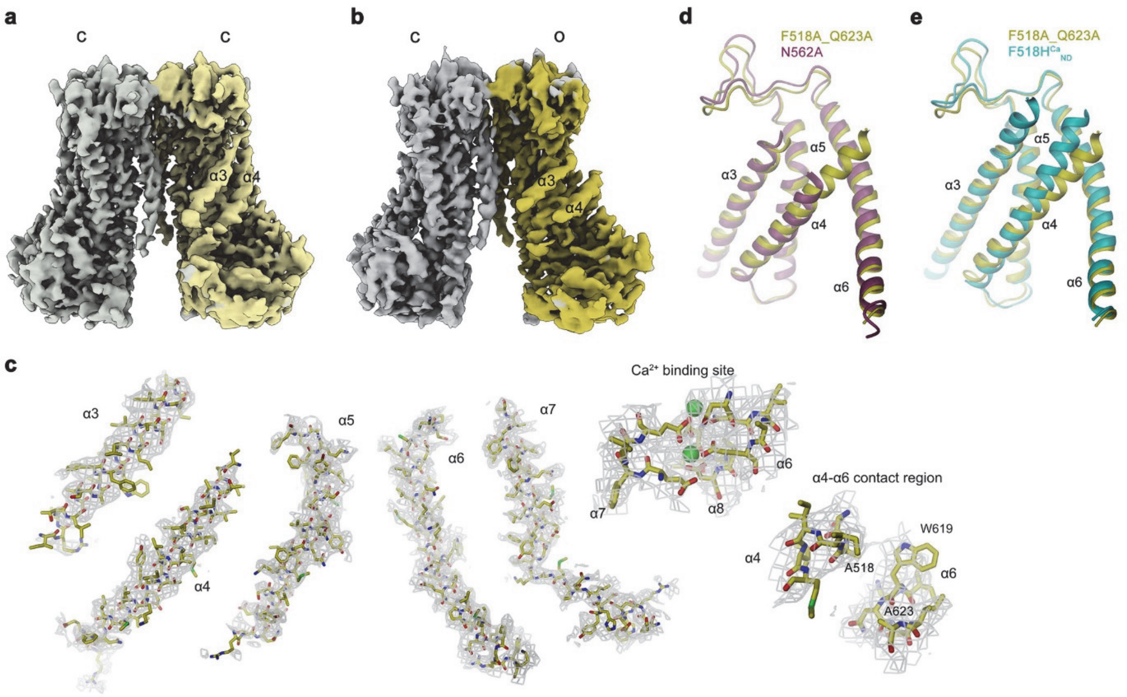

Supplementary Fig. 10: Structural features of F518A_Q623A^{Ca}. a, b Cryo-EM density maps of **a**, the symmetric population of F518A_Q623A$^{Ca}_{cc}$ with both subunits residing in a closed (c) state and **b**, the asymmetric population of F518A_Q623A$^{Ca}_{co}$, with one subunit residing in the closed (c) and the other in the open (o) state. The subunit adopting the activated conformation is shown in color. **c**, Cryo-EM densities of the protein in an open conformation (contoured at 5.5 σ for α3 and α4 and at 6.5 σ for all other regions) superimposed on selected parts of the model. **d, e** Superposition of the open states of F518A_Q623A^{Ca} on **d**, N562A^{Ca} and **e**, F518H$^{Ca}_{ND}$. α-helices lining the subunit cavity are labeled and shown as ribbon.

Supplementary Table 1.

	F518A_Q623A$^{Ca}{}_{OC}$ (EMD-15958) (PDB 8BC0))	F518A_Q623A$^{Ca}{}_{CC}$ (EMD-15959) (PDB 8BC1)
**Data collection and processing**		
Magnification	130.000	130.000
Voltage (kV)	300	300
Electron exposure (e–/Å^2)	59.46	59.46
Defocus range (μm)	-1 to -2.4	-1 to -2.4
Pixel size (Å)	1.302	1.302
Movies recorded	10,605	10,605
Symmetry imposed	C1	C2
Initial particle images (no.)	2,373,759	2,373,759
Final particle images (no.)	208,801	183,302
Map resolution (Å)	3.09	3.01
FSC threshold	0.143	0.143
Map resolution range (Å)	2.9-6.0	2.9-6.0
**Refinement**		
Initial model used (PDB code)	6QP6	6QPB
Model resolution (Å)	3.8	4.2
FSC threshold	0.5	0.5
Map sharpening *B* factor (Å^2)	69.7	75.8
Model vs. Map CC (mask)	0.6	0.54
Model composition		
Non-hydrogen atoms	10861	8684
Protein residues	1316	1054
Ligands	6 Ca^{2+}	6 Ca^{2+}
B factors (Å^2)		
Protein	100.37	1100.19
Ligand	96.63	91.51
R.m.s. deviations		
Bond lengths (Å)	0.004	0.003
Bond angles (°)	0.586	0.520
Validation		
MolProbity score	2.73	2.44
Clashscore	17.1	12.46
Poor rotamers (%)	7.06	4.82
Ramachandran plot		
Favored (%)	94.98	95.52
Allowed (%)	5.02	24.48
Disallowed (%)	0.00	0.00

Supplementary Table 2: List of primers.

Name	F/R	Sequence (5'-to-3')
S510A	F	TGG CCA CAG CCA TCA CAG CCT CCA TCA TCA GCT TCA TCA TCA TCA TGA TC
S510A	R	GCT GTG ATG GCT GTG GCC ATC TGT GGG GTC AGG TAC TTC TGG
I511A	F	GCC ACA TCC GCC ACA GCC TCC ATC ATC AGC TTC ATC ATC
I511A	R	GGA GGC TGT GGC GGA TGT GGC CAT CTG TGG GGT CAG GTA
I515A	F	ACA GCC TCC GCC ATC AGC TTC ATC ATC ATC ATG ATC CTC
I515A	R	GAA GCT GAT GGC GGA GGC TGT GAT GGA TGT GGC CAT CTG
F518A	F	CCA TCA TCA GCG CCA TCA TCA TCA TGA TCC TCA ACA CGA TCT ACG AGA AGG
F518A	R	ATG ATG ATG ATG GCG CTG ATG ATG GAG GCT GTG ATG GAT GTG GCC
F519Y	F	CCT CCA TCA TCA GCT ACA TCA TCA TCA TGA TCC TCA ACA CGA TCT ACG AGA AGG T
F518Y	R	ATC ATG ATG ATG ATG TAG CTG ATG ATG GAG GCT GTG ATG GAT GTG GCC ATC TGT G
F518Q	F	ATC ATC AGC CAA ATC ATC ATC ATG ATC CTC AAC ACG ATC
F518Q	R	GAT GAT GAT TTG GCT GAT GAT GGA GGC TGT GAT GGA TGT
F518H	F	ATC ATC AGC CAC ATC ATC ATC ATG ATC CTC AAC ACG ATC
F518H	R	GAT GAT GAT GTG GCT GAT GAT GGA GGC TGT GAT GGA TGT
I521A	F	TTC ATC ATC GCC ATG ATC CTC AAC ACG ATC TAC GAG AAG
I521A	R	GAG GAT CAT GGC GAT GAT GAA GCT GAT GAT GGA GGC TGT
M522A	F	ATC ATC ATC GCG ATC CTC AAC ACG ATC TAC GAG AAG GTG
M522A	R	GTT GAG GAT CGC GAT GAT GAT GAA GCT GAT GAT GGA GGC
L524A	F	ATC ATG ATC GCC AAC ACG ATC TAC GAG AAG GTG GCC ATC
L524A	R	GAT CGT GTT GGC GAT CAT GAT GAT GAT GAA GCT GAT GAT
L558A	F	ATG TTC TTG GCC CAG TTT GTC AAC TAT TAC TCC TCA TGC
L558A	R	GAC AAA CTG GGC CAA GAA CAT CTT CAT GGT CAG GCT GTT
Q559A	F	TTG TTC GCA TTT GTC AAC TAT TAC TCC TCA TGC TTC TAC
Q559A	R	GAC AAA TGC GAA CAA GAA CAT CTT CAT GGT CAG GCT GTT
N562A	F	TTT GTC GCA TAT TAC TCC TCA TGC TTC TAC ATC GCA TTC
N562A	R	GTA ATA TGC GAC AAA CTG GAA CAA GAA CAT CTT CAT GGT
Y563A	F	TTT GTC AAC GCT TAC TCC TCA TGC TTC TAC ATC GCA TTC
Y563A	R	TGA GGA GTA AGC GTT GAC AAA CTG GAA CAA GAA CAT CTT
Y563H	F	TTT GTC AAC CAT TAC TCC TCA TGC TTC TAC ATC GCA TTC
Y563H	R	TGA GGA GTA ATG GTT GAC AAA CTG GAA CAA GAA CAT CTT
C567A	F	TCC TCA GCT TTC TAC ATC GCA TTC TTC AAG GGC AAA TTC

C567A	R	GTA GAA AGC TGA GGA GTA ATA GTT GAC AAA CTG GAA CAA
I570A	F	TTC TAC GCT GCA TTC TTC AAG GGC AAA TTC GTG GGC TAT
I570A	R	GAA TGC AGC GTA GAA GCA TGA GGA GTA ATA GTT GAC AAA
E604A	F	CTG CCT CCT TGC ACT GAC CAC ACA GCT GAC GAT CAT CAT GGG G
E604A	R	GTG GTC AGT GCA AGG AGG CAG CCC CCC GGG TCA CAC TCT
Q608A	F	CTG ACC ACA GCG CTG ACG ATC ATC ATG GGG GGA AAG GCA ATC T
Q608A	R	TCG TCA GCG CTG TGG TCA GTT CAA GGA GGC AGC CCC CC
L609A	F	CAC ACA GGC TAC GAT CAT CAT GGG GGG AAA GGC AAT CTG G
L609A	R	GAT CGT AGC CTG TGT GGT CAG TTC AAG GAG GCA GCC CCC
I611A	F	CAG CTG ACG GCC ATC ATG GGG GGA AAG GCA ATC TGG AAC
I611A	R	CCC CAT GAT GGC CGT CAG CTG TGT GGT CAG TTC AAG GAG
I612A	F	CTG ACG ATC GCC ATG GGG GGA AAG GCA ATC TGG AAC AAC ATA CAA GAA G
I612A	R	CCC CCA TGG CGA TCG TCA GCT GTG TGG TCA GTT CAA GGA GGC
K616A	F	GGG GGG AGC GGC AAT CTG GAA CAA CAT ACA AGA AGT CTT GCT CCC A
K616A	R	CAG ATT GCC GCT CCC CCC ATG ATG ATC GTC AGC TGT GTG GTC
W619A	F	GAA AGG CAA TCG CGA ACA ACA TAC AAG AAG TCT TGC TCC CAT GGG TTA TGA ATC
W619A	R	TTG TAT GTT GTT CGC GAT TGC CTT TCC CCC CAT GAT GAT CGT CAG CTG
Q623A	F	AAC ATA GCT GAA GTC TTG CTC CCA TGG GTT ATG AAT CTA
Q623A	R	GAC TTC AGC TAT GTT GTT CCA GAT TGC CTT TCC CCC CAT
G473A	F	GCC TCT GTG ATC GCG ATC ATT GTC TAC AGG CTG TCC GTG TTC ATC GTA
G473A	R	AGA CAA TGA TCG CGA TCA CAG AGG CGA TGA TGA GCA GGA TCC AGA AGA AGA CG
G473P	F	GCC TCT GTG ATC CCG ATC ATT GTC TAC AGG CTG TCC GTG TTC ATC GTA
G473P	R	AGA CAA TGA TCG GGA TCA CAG AGG CGA TGA TGA GCA GGA TCC AGA AGA AGA CG

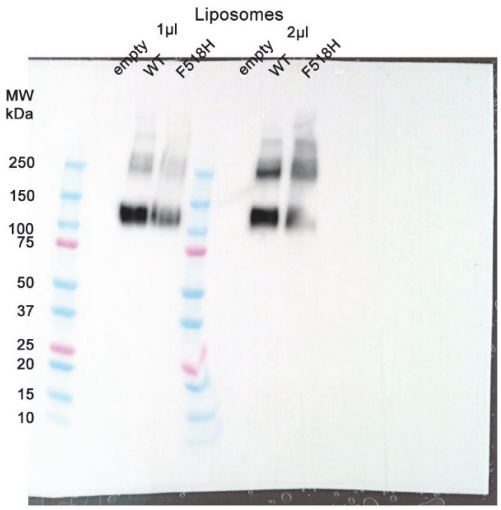

Western Blot for Supplementary Fig. 2a
