# Supplementary material for: Structural basis for the activation of the lipid scramblase TMEM16F
Source: Nat Commun. 2022 Nov 5;13:6692. doi: 10.1038/s41467-022-34497-x (PMC9637102; doi:10.1038/s41467-022-34497-x)
Supplement: Supplementary file 3 — Description of Additional Supplementary Files [file 41467_2022_34497_MOESM3_ESM.pdf]

File name: Supplementary Movie 1

Description: Morph between known TMEM16F structures defining a pathway towards activation. The video shows a single subunit in two different orientations. The protein is displayed as ribbon, with selected α-helices shown in unique colors (α3 red, α4 green, α6 blue). Cα positions of F518, W619 and Q623, which interact in the active conformation, are shown as red spheres. Membrane boundaries are indicated.
