# Supplementary material for: Structural basis for the activation of the lipid scramblase TMEM16F
Source: Nat Commun. 2022 Nov 5;13:6692. doi: 10.1038/s41467-022-34497-x (PMC9637102; doi:10.1038/s41467-022-34497-x)
Supplement: Supplementary file 5 — Reporting Summary [file 41467_2022_34497_MOESM5_ESM.pdf]

nature research

Corresponding author(s): Raimund Dutzler

Last updated by author(s): Oct 14, 2022

Reporting Summary

Nature Research wishes to improve the reproducibility of the work that we publish. This form provides structure for consistency and transparency in reporting. For further information on Nature Research policies, see our Editorial Policies and the Editorial Policy Checklist.

Statistics

For all statistical analyses, confirm that the following items are present in the figure legend, table legend, main text, or Methods section.

n/a	Confirmed	
☐	☒	The exact sample size (*n*) for each experimental group/condition, given as a discrete number and unit of measurement
☐	☒	A statement on whether measurements were taken from distinct samples or whether the same sample was measured repeatedly
☒	☐	The statistical test(s) used AND whether they are one- or two-sided<br>*Only common tests should be described solely by name; describe more complex techniques in the Methods section.*
☒	☐	A description of all covariates tested
☒	☐	A description of any assumptions or corrections, such as tests of normality and adjustment for multiple comparisons
☐	☒	A full description of the statistical parameters including central tendency (e.g. means) or other basic estimates (e.g. regression coefficient) AND variation (e.g. standard deviation) or associated estimates of uncertainty (e.g. confidence intervals)
☒	☐	For null hypothesis testing, the test statistic (e.g. *F*, *t*, *r*) with confidence intervals, effect sizes, degrees of freedom and *P* value noted<br>*Give P values as exact values whenever suitable.*
☒	☐	For Bayesian analysis, information on the choice of priors and Markov chain Monte Carlo settings
☒	☐	For hierarchical and complex designs, identification of the appropriate level for tests and full reporting of outcomes
☒	☐	Estimates of effect sizes (e.g. Cohen's *d*, Pearson's *r*), indicating how they were calculated

Our web collection on statistics for biologists contains articles on many of the points above.

Software and code

Policy information about availability of computer code

Data collection	Clampex 10.7, EPU 2.9
Data analysis	Clampfit 10.7, Excel 2019, Fiji, GraphPad Prism 9, cryoSPARC v. 2.7-3.1, Phenix 1.19.2, Coot 0.9.5, HOLE 2.2.005, UCSF Chimera 1.15, ChimeraX 1.2.5, Dino, MSMS

For manuscripts utilizing custom algorithms or software that are central to the research but not yet described in published literature, software must be made available to editors and reviewers. We strongly encourage code deposition in a community repository (e.g. GitHub). See the Nature Research guidelines for submitting code & software for further information.

Data

Policy information about availability of data

All manuscripts must include a data availability statement. This statement should provide the following information, where applicable:

- Accession codes, unique identifiers, or web links for publicly available datasets
- A list of figures that have associated raw data
- A description of any restrictions on data availability

The cryo-EM maps, half-maps, and masks have been deposited in the Electron Microscopy Data Bank under accession numbers EMD-15913 (F518HnoCa), EMD-15914
(F518HCa), EMD-15919 (F518HCaND), EMD-15917 (N562ACaOC), EMD-15916 (N562ACaCC). Coordinates for models are available in the Protein Data Bank under PDBIDs 8B8G (F518HnoCa), 8B8J (F518HCa), 8B8Q (F518HCaND), 8B8M (N562ACaOC), 8B8L (N562ACaCC).

The study has also relied on the following datasets deposited with the PDB:
PDBID 6QPB P[http://doi.org/10.2210/pdb6QPB/pdb]

PDBID 6QP6 [http://doi.org/10.2210/pdb6QP6/pdb]
PDBID 6QPC [http://doi.org/10.2210/pdb6QPC/pdb]

Source data are provided with this paper. Primary electrophysiology recordings and lipid scrambling traces supporting the findings of this study are numerous and thus available from the corresponding authors upon reasonable request.

Field-specific reporting

Please select the one below that is the best fit for your research. If you are not sure, read the appropriate sections before making your selection.

☒ Life sciences ☐ Behavioural & social sciences ☐ Ecological, evolutionary & environmental sciences

For a reference copy of the document with all sections, see nature.com/documents/nr-reporting-summary-flat.pdf

Life sciences study design

All studies must disclose on these points even when the disclosure is negative.

Sample size	No sample size determination was performed. Experiments were performed multiple times with similar results and further inclusion of data did not change the results.
Data exclusions	Leaky recordings were discarded, and otherwise no data were excluded from the analyses
Replication	Electrophysiology experiments were repeated multiple times and the number of biological replicates and errors are indicated. All attempts at replication were successful.
Randomization	Not applicable, randomization is not relevant.
Blinding	Not applicable, blinding is not relevant.

Reporting for specific materials, systems and methods

We require information from authors about some types of materials, experimental systems and methods used in many studies. Here, indicate whether each material, system or method listed is relevant to your study. If you are not sure if a list item applies to your research, read the appropriate section before selecting a response.

Materials & experimental systems

n/a	Involved in the study	
☐	☒	Antibodies
☐	☒	Eukaryotic cell lines
☒	☐	Palaeontology and archaeology
☒	☐	Animals and other organisms
☒	☐	Human research participants
☒	☐	Clinical data
☒	☐	Dual use research of concern

Methods

n/a	Involved in the study	
☒	☐	ChIP-seq
☒	☐	Flow cytometry
☒	☐	MRI-based neuroimaging

Antibodies

Antibodies used	Peroxidase-conjugated AffiniPure Goat Anti-Mouse IgG Jackson Immuno Research cat#115035003 polyclonal, MONOCLONAL ANTI-c-MYC<br>Purified Mouse Immunoglobulin SIGMA cat#M4439 clone 9E10
Validation	These are widely used commercial antibodies against fusion tags which have been validated on overexpressed and purified protein. This monoclonal anti-myc tag Antibody was previosuly verified by Western Blot using purified myc-tagged protein reconstituted into proteoliposomes. Alvadia, C., Lim, N.K., Clerico Mosina, V., Oostergetel, G.T., Dutzler R.* & Paulino, C.* Cryo-EM structures and functional characterization of the murine lipid scramblase TMEM16F. Elife 8, doi:10.7554/eLife.44365 (2019).

Eukaryotic cell lines

Policy information about cell lines

Cell line source(s)	HEK293T (ATCC CRL-1573) , HEK293 TMEM16F-/- (Le, T., Le, S. C. & Yang, H. Drosophila Subdued is a moonlighting transmembrane protein 16 (TMEM16) that transports ions and phospholipids. The Journal of biological chemistry 294, 4529-4537, doi:10.1074/jbc.AC118.006530 (2019))<br>Murine TMEM16F inducible stable HEK 293 cell line generated from a Flp-In T-REx293 cell line (ThermoFischer cat#R78007)

described in Alvadia, C. et al. Cryo-EM structures and functional characterization of the murine lipid scramblase TMEM16F. Elife 8, doi:10.7554/eLife.44365 (2019).

Authentication	For commercially available cell lines, no further cell line authentication was performed. For the TMEM16F-/- cell-line provided by Prof. Huanghe Yang, we did not find and Ca2+-dependent scrambling response in cellular assays. The genetic knockout fro this cell-line was previously confirmed on a DNA level . (Le, T., Le, S. C. & Yang, H. Drosophila Subdued is a moonlighting transmembrane protein 16 (TMEM16) that transports ions and phospholipids. The Journal of biological chemistry 294, 4529-4537, doi:10.1074/jbc.AC118.006530 (2019))
Mycoplasma contamination	The cell lines were tested and are free from mycoplasma contamination.
Commonly misidentified lines (See ICLAC register)	No commonly misidentified cell lines were used in the study
